# Decoding adolescent TMJ osteoarthritis with multimodal machine learning

**DOI:** 10.22514/jofph.2026.021

**Published:** 2026-03-12

**Authors:** Yeon-Hee Lee, Do-Hoon Kim, Akhilanand Chaurasia, Tae-Seok Kim, Fernando P.S. Guastaldi, Yung-Kyun Noh

**Affiliations:** ^1^Department of Orofacial Pain and Oral Medicine, College of Dentistry, Kyung Hee University Dental Hospital, Kyung Hee University, 02447 Seoul, Republic of Korea; ^2^Center for Systems Biology, Massachusetts General Hospital, Harvard Medical School, Boston, MA 02114, USA; ^3^Department of Computer Science, Hanyang University, 04763 Seoul, Republic of Korea; ^4^Department of Oral Medicine and Radiology, King George’s Medical University, 226003 Lucknow, India; ^5^Division of Oral and Maxillofacial Surgery, Department of Surgery, Massachusetts General Hospital, Harvard School of Dental Medicine, Boston, MA 02114, USA; ^6^School of Computational Sciences, Korea Institute for Advanced Study (KIAS), 02455 Seoul, Republic of Korea

**Keywords:** Temporomandibular disorders, Osteoarthritis, Adolescents, Magnetic resonance imaging, Panoramic radiography, Machine learning, Decision trees

## Abstract

**Background**: Early and accurate diagnosis of adolescent temporomandibular joint (TMJ) osteoarthritis (OA) is critical, as degenerative changes during growth can cause lifelong pain and deformity. This study aimed to identify key clinical and imaging predictors of adolescent TMJ-OA and to evaluate multimodal machine learning models. **Methods**: The diagnostic utility was evaluated in 79 adolescents (10–18 years) with TMJ pain using panoramic radiography (PR) and MRI. TMJ-OA was diagnosed based on the Diagnostic Criteria for Temporomandibular Disorders (DC/TMD). Three decision tree models were developed: Model 1 (clinical-only), Model 2 (imaging-only), and Model 3 (combined clinical and imaging). Logistic regression was used for the comparisons. **Results**: To ensure a robust evaluation with a small sample size (n = 79), the models were assessed using nested 5-fold cross-validation. Model 2 (imaging only) had the highest specificity (0.7714 ± 0.2321), accuracy (0.5942 ± 0.0966), and AUROC (0.719 ± 0.101), but a low sensitivity (0.4472 ± 0.2065). PR evidence of TMJ-OA (feature importance = 0.70; OR = 3.93) was the strongest predictor and root node in the decision tree. Model 3 (combined clinical and imaging data) showed improved sensitivity (0.6056 ± 0.1829), identifying PR_TMJ_OA, MRI_TMJ_ADD (anterior disc displacement), Visual Analog Scale (VAS) score, and age as key nodes (AUROC = 0.6573 ± 0.0338; OR = 2.85 for PR_TMJ_OA). Model 1 (clinical-only) had limited predictive performance (AUROC = 0.4859 ± 0.0894), with symptom duration (importance = 0.64; OR = 1.40), VAS score, and joint locking (importance = 0.20) contributing modestly. A model using PR_TMJ_OA alone achieved perfect specificity (0.9714 ± 0.0571) but low sensitivity (0.3806 ± 0.1458). **Conclusions**: Although PR is a meaningful screening tool for adolescent TMJ-OA, it remains insufficient as a standalone diagnostic modality. Multimodal integration of clinical and MRI findings improves diagnostic accuracy and provides interpretable, clinically aligned decision-support tools for TMJ-OA.

## 1. Introduction

Temporomandibular joint osteoarthritis (TMJ-OA) can emerge during adolescence, a period of active craniofacial growth, during which early degenerative changes may have disproportionately long-term consequences. Unlike transient forms of temporomandibular disorders (TMDs), early onset TMJ-OA may progress to irreversible skeletal deformities, persistent orofacial pain, and functional impairment, extending into adulthood [1]. TMD symptoms affect 6–68% of adolescents [2]; although many cases are mild, a subset progresses to joint pathology, underscoring the importance of early recognition and timely intervention.

Diagnostic frameworks such as the Research Diagnostic Criteria for Temporomandibular Disorders (RDC/TMD) and the updated Diagnostic Criteria for Temporomandibular Disorders (DC/TMD) define TMJ-OA primarily based on clinical findings, including TMJ pain with crepitus, but emphasize that imaging is required for confirmation [3, 4]. Recent interdisciplinary consensus guidelines for juvenile idiopathic arthritis have highlighted the importance of accurately diagnosing TMJ involvement [5]. Conventional panoramic radiography (PR) is widely used for screening because of its accessibility and low radiation burden, whereas cone-beam computed tomography (CBCT) provides high-resolution visualization of osseous changes and is regarded as the gold standard imaging modality [6]. However, early-stage OA in adolescents predominantly involves bone marrow signal alterations, inflammation, and soft tissue abnormalities, which are not detectable by CBCT [7]. Magnetic resonance imaging (MRI) has been increasingly adopted in recent OA studies as the reference standard for identifying early degenerative changes [8]. MRI also provides a complementary assessment of osseous and soft tissues, enabling evaluation of disc displacement and early degenerative changes [9]. However, CBCT is less practical for adolescents because of the higher radiation exposure and cost, whereas MRI, despite its diagnostic advantages, is limited by accessibility and expense [10]. Therefore, PR remains the most feasible first-line imaging modality for adolescents, although its diagnostic accuracy has been questioned. Clinical symptoms alone are nonspecific, and radiographic findings may underestimate or overestimate disease severity [11]. Thus, reliable diagnosis requires integrating both clinical and imaging information; however, few studies have systematically applied multimodal approaches to adolescent cohorts.

Machine learning (ML) provides a framework. Among the available algorithms, decision tree models are particularly advantageous because they mirror the stepwise reasoning of clinical judgment, assign relative weights to predictors, and provide interpretable outputs aligned with diagnostic decision-making [12]. Logistic regression complements ML analyses by providing effect size estimates and enhancing their interpretability [13]. This transparency distinguishes them from “black-box” models and increases their clinical applicability.

Research applying multimodal ML to adolescent TMJ-OA remains scarce, despite its clinical importance. Accordingly, the present study aimed to identify the clinical and imaging factors associated with MRI-confirmed TMJ-OA in adolescents and to develop interpretable multimodal ML models. By comparing models based on clinical features, imaging features, and their integration, we sought to clarify the diagnostic value of each and determine whether PR alone could serve as a reliable screening tool for TMJ-OA. We hypothesized that (1) clinical variables alone would be insufficient, (2) PR would demonstrate high sensitivity but low specificity, and (3) optimal prediction would require a combination of clinical and imaging features.

## 2. Materials and methods

### 2.1 Study population and groups

This retrospective study enrolled 79 adolescents (50 females and 29 males; mean age, 15.2 ± 2.2 years) who presented with TMD and TMJ pain between January 2023 and August 2024. The patients were initially categorized into TMJ-OA and non-TMJ-OA groups according to the Diagnostic Criteria for TMD (DC/TMD) by two calibrated examiners (LYH and TSK), both specialists in orofacial pain and TMD [3]. Clinically, this requires pain localized to the TMJ, as confirmed by palpation or jaw movement, in combination with crepitus. Imaging evidence of degenerative osseous changes (erosion, osteophytes, flattening, or sclerosis) on PR or MRI was used to confirm the diagnosis. The inter- and intra-examiner reliabilities were excellent (Cohen kappa >0.83), with consistently high diagnostic agreement across all the evaluated variables. Any discrepancies were resolved by consensus agreement. In this study, MRI findings were considered the gold standard for confirming a TMJ-OA diagnosis, whereas PR findings were evaluated against MRI results. The inclusion criteria were as follows: age of 10–18 years, clinical symptoms of TMD with TMJ pain, bilateral MRI and PR at assessment, and informed consent from the patient or guardian.

The exclusion criteria included a history of TMJ surgery or systemic joint disease (*e.g.*, juvenile idiopathic arthritis or rheumatoid arthritis), prior fracture injury or orofacial surgery, presence of severe degenerative disease or congenital craniofacial anomalies causing major skeletal deformities that could confound TMJ morphology assessment (*e.g.*, cleft lip/palate, craniosynostosis, and genetic syndromes), severe cognitive disorders precluding the completion of questionnaires, and incomplete clinical or psychological data.

### 2.2 Clinical data collection

Clinical variables were obtained during the initial visit using standardized DC/TMD protocols. The data included pain intensity on a visual analog scale (VAS, 0–10), symptom duration, pain laterality (unilateral or bilateral), bruxism, and TMJ-related symptoms such as joint noises, muscle stiffness, and locking [14].

### 2.3 Panoramic radiography

PR was performed during the first visit under standardized exposure settings (70 kVp, 12 mA). TMJ osteoarthritis was diagnosed on panoramic radiography when one or more of the following primary radiographic features were present: erosion, indistinct cortical bone outline, osteophyte formation, or subchondral cysts. These findings can occur with or without secondary changes, such as condylar flattening or subcortical sclerosis [15]. In addition to degenerative changes, skeletal discrepancies were evaluated to explore potential craniofacial associations with TMJ pathology. Nasion-maxilla discrepancy (Na-Mx) was defined as a misalignment between the vertical line passing through the nasion and the central axis of the maxilla, whereas maxilla-mandible discrepancy (Mx-Mn) was defined as a lack of overlap between the central axes of the maxilla and mandible (Fig. 1).

**Fig. 1.** Bilateral TMJ-OA observed on MRI and panoramic radiography in a 17-year-old female patient. (A) T1-weighted MRI of the right temporomandibular joint (TMJ) demonstrating anterior disc displacement (ADD) accompanied by condylar degeneration. (B) MRI of the left TMJ showing an ADD with erosion of the cortical bone lining. Yellow arrow: anterior disc displacement; red arrowheads: condylar degeneration. (C) Panoramic radiograph illustrating skeletal reference lines. Na (nasion line, white dashed line) indicates the midsagittal reference line; Mx (maxilla line, red dashed line) and Mn (mandible line, green dashed line) represent the midline axes of the maxilla and mandible, respectively. R: right side.

### 2.4 MRI acquisition and interpretation

MRI was performed using a 3.0 T system (Signa Genesis; GE Healthcare, Waukesha, WI, USA) with a 6 × 8 cm TMJ surface coil. The sequences included T1-weighted, T2-weighted, and proton density imaging in the sagittal and coronal planes, with a slice thickness of ≤3 mm. A 15 cm field of view and a 256 × 224 matrix were used to optimize spatial resolution and image quality. Disc position was evaluated in both closed and open mouth positions, and TMJ-OA was defined as disc deformity and/or condylar degeneration. Anterior disc displacement (ADD) was recorded for each joint [14]. TMJ-OA and ADD findings could coexist and were assessed independently. The results were used for analyses based on laterality (unilateral *vs.* bilateral), side-specific comparisons, and person-level assessments (Fig. 1).

### 2.5 Machine learning model development and evaluation

To mitigate the instability associated with a single train/test split evaluation, particularly given the limited sample size (n = 79), we implemented Nested 5-Fold Cross-Validation (NCV) with both the outer and inner folds set to K = 5. This NCV framework separates model evaluation on independent test sets (outer folds) from hyperparameter tuning on the training sets (inner folds). During the NCV process, the dataset (n = 79) was partitioned into training (80%) and testing (20%) subsets using stratified sampling to maintain the original class distribution. Two models were implemented: logistic regression and decision tree classification. To address class imbalance, a synthetic minority oversampling technique (SMOTE) was applied [16]. Although our dataset was not severely imbalanced (44 TMJ-OA patients *vs.* 35 non-TMJ-OA patients), SMOTE was applied only within the inner training folds of the NCV [17]. This ensured a balanced 50:50 class distribution during training by oversampling the minority “non-TMJ-OA” classes. Logistic regression coefficients were used to estimate the odds ratios (OR) for each predictor, and decision tree feature importance was derived from Gini impurity reduction. Hyperparameters were optimized using stratified 5-fold cross-validation to enhance model generalizability and reduce overfitting. The model performance was assessed using AUROC, accuracy, sensitivity, specificity, and F1-score. Receiver Operating Characteristic (ROC) curves were plotted to illustrate the discriminative ability. The complete source code used for the model development and analysis is publicly available at https://github.com/Dohoon1/TMJ_OA/tree/main/.

### 2.6 Statistical analysis

All statistical analyses were performed using SPSS for Windows (version 25.0; IBM Corp., Armonk, NY, USA), R software (version 4.0.2; R Foundation for Statistical Computing), and Python software (version 3.9.7; Python Software Foundation). Continuous variables were compared using *t* tests, and categorical variables were compared using the chi-square or Fisher’s exact tests, as appropriate. Inter-rater reliability was quantified using Cohen’s kappa, and effect sizes were calculated for the significant findings. Logistic regression analyses were used to identify the predictors of TMJ-OA, and the results were reported as odds ratios (ORs) with 95% confidence intervals. The performance of the three decision tree and logistic regression models was compared with the results from the five outer test folds of the NCV. This approach offers a robust estimate of generalizability and quantitatively reflects the variability in model performance across different data splits. DeLong’s test was used to compare the AUROC values of the models. Statistical significance was set at *p * < 0.05.

## 3. Results

### 3.1 Demographic characteristics

Among the 79 adolescents with TMJ pain, 55.7% were diagnosed with TMJ, and 79.7% had ADD. Patients were divided into non-TMJ-OA (n = 35; mean age 15.5 ± 2.0 years; 23 females, 12 males) and TMJ-OA (n = 44; mean age 15.3 ± 2.0 years; 27 females, 17 males) groups. The age and sex distributions did not differ significantly (*p* = 0.799 and *p* = 0.815, respectively). Overall, females were predominant (female-to-male ratio, 1.85:1); however, no sex-specific association with OA was observed (Table 1).

**Table 1.** Demographic and clinical characteristics.

Parameter		Non-TMJ-OA (n = 35) mean ± SD or n (%)	TMJ-OA (n = 44) mean ± SD or n (%)	*p* value
Demographics				
	Age (yr)^a^	15.46 ± 1.98	15.34 ± 2.03	0.799
	Sex^b^			
	- Male	12 (34.3%)	17 (38.6%)	0.815
	- Female	23 (65.7%)	27 (61.4%)	
Pain characteristics				
	VAS (0–10)^a^	3.00 ± 2.09	3.52 ± 2.19	0.286
	Symptom duration (mon)^a^	11.91 ± 16.27	15.00 ± 19.68	0.458
	Symptom chronicity^b^	19 (54.3%)	23 (52.3%)	1.000
Pain side^b^				
	Right	14 (40.0%)	12 (27.3%)	0.481
	Left	8 (22.9%)	13 (29.5%)	
	Bilateral	13 (37.1%)	19 (43.2%)	
Chief complaint with TMJ arthralgia				
	TMJ noise^b^			
	- (Absence)	11 (31.4%)	16 (36.4%)	0.812
	- (Presence)	24 (68.6%)	28 (63.6%)	
	Muscle stiffness^b^			
	- (Absence)	16 (45.7%)	25 (56.8%)	0.370
	- (Presence)	19 (54.3%)	19 (43.2%)	
	Locking^b^			
	- (Absence)	13 (37.1%)	18 (40.9%)	0.818
	- (Presence)	22 (62.9%)	26 (59.1%)	
	Bruxism^b^			
	- (Absence)	26 (74.3%)	29 (65.9%)	0.468
	- (Presence)	9 (25.7%)	15 (34.1%)	

*^a^Results were obtained using a t-test adjusted for Bonferroni correction. ^b^Results were analyzed using a χ^2^ test between two age groups with Bonferroni adjustment. To obtain significant results, the two-tailed level of statistical significance was set at p < 0.05. TMJ: temporomandibular joint; OA: osteoarthritis; SD: standard deviation; VAS: visual analogue scale.*

### 3.2 Pain characteristics

The TMJ-OA group reported a longer mean symptom duration (15.0 ± 19.7 months *vs.* 11.9 ± 16.3 months) and higher VAS pain scores (3.5 ± 2.2 *vs.* 3.0 ± 2.1) than the non-OA group, although the differences were not statistically significant. Chronicity (≥6 months) was present in approximately half of the patients in both groups (52.3% *vs.* 54.3%). The laterality of symptoms also did not show any significant group differences. Unilateral pain was more common in both groups (56.8% in the OA group and 62.9% in the non-OA group), with a right-sided predominance in patients without OA. The detailed laterality data are summarized in **Supplementary Table 1**.

### 3.3 Chief complaint

The most frequent symptoms in both groups were TMJ noise, jaw locking, muscle stiffness, and bruxism. The frequency of each complaint did not differ significantly between OA and non-OA patients (all *p * > 0.3) (Table 1).

### 3.4 MRI findings

MRI revealed a unilateral-to-bilateral TMJ-OA ratio of 2.39:1, with left-sided involvement more frequent (40.9%) than right-sided involvement (29.5%), and 29.5% of patients had bilateral OA. ADD was observed in 79.7% of all patients and was significantly more prevalent in the TMJ-OA group (86.4% *vs.* 71.4%). Bilateral ADD was particularly common in patients with OA (70.5% *vs.* 34.3%, *p* = 0.015). Left-sided ADD was also more frequent in patients with OA (79.5% *vs.* 51.4%, *p* = 0.015), whereas right-sided ADD showed a non-significant trend (77.3% *vs.* 54.3%, *p* = 0.053). The complete laterality distributions are provided in **Supplementary Table 2**.

### 3.5 Panoramic radiography findings

Radiographic evidence of OA on PR was detected in 61.4% of patients with OA. Among them, unilateral PR OA was observed on the right side in 6.8% and on the left side in 13.6%, with bilateral PR OA in 18.2% of patients. Notably, skeletal discrepancies were frequent but not group-specific: Na-Mx discrepancy occurred in 63.6% of OA and 65.7% of non-OA patients (*p* = 1.0), whereas Mx-Mn discrepancy was present in 36.4% *vs.* 40.4% of non-OA patients (*p* = 0.817). Overall, 64.6% of the cohort exhibited Na-Mx discrepancies, and 37.9% exhibited Mx-Mn discrepancies.

### 3.6 Predictive value of PR

PR-detected OA was strongly associated with MRI-confirmed OA (*p * < 0.001). A simple decision tree model using panoramic radiography (PR) in diagnosing temporomandibular joint (TMJ) osteoarthritis (OA) (PR_TMJ_OA) as the sole predictor achieved a specificity of 0.9714 ± 0.0571 but low sensitivity (0.3806 ± 0.1458), with AUROC = 0.6760 ± 0.0722 and overall accuracy of 0.6442 ± 0.0722. The corresponding tree structure is presented in **Supplementary Fig. 1**.

### 3.7 Correlation analysis

The correlation matrices demonstrated a moderately positive relationship between MRI-confirmed and PR-detected OA (*r* = 0.37, *p* = 0.001). No significant association was found between TMJ-OA and skeletal discrepancies. Side-specific analyses confirmed moderate correlations between MRI OA and PR OA on both the right (*r* = 0.53, *p * < 0.001) and left (*r* = 0.51, *p * < 0.001) sides, with weaker correlations between OA and ADD. The full correlation matrices are provided in **Supplementary Fig. 2**. Bruxism was moderately correlated with the Mx-Mn discrepancy (*r* = 0.28, *p* = 0.012).

### 3.8 Decision tree and logistic regression models

To clarify the contributions of clinical and imaging variables in predicting MRI-confirmed TMJ-OA, correlations among key features were examined. MRI-confirmed OA showed a moderately positive correlation with PR-detected OA (*r* = 0.42, *p * < 0.001). MRI-detected ADD showed a weak but statistically significant association with symptom duration (*r* = 0.20, *p* = 0.039). Bruxism exhibited a moderate correlation with Mx-Mn skeletal discrepancy (*r* = 0.28, *p* = 0.012), indicating a potential interaction between occlusal relationships and parafunctional habits.

Based on these findings, three predictive models were constructed using distinct feature sets: Model 1 (clinical-only), Model 2 (imaging-only), and Model 3 (combined clinical and imaging data). Each model was evaluated using decision tree classification and logistic regression to compare the interpretability, dominant predictors, and diagnostic performance (Table 2). The model performance based on Nested 5-Fold Cross-Validation is summarized in **Supplementary Tables 3 and 4**.

**Table 2.** Prediction performance of each decision tree and logistic regression model.

Parameter	Model 1 (only clinical data)		Model 2 (only imaging data)		Model 3 (clinical + imaging data)		*p* value
	Decision tree	Logistic regression	Decision tree	Logistic regression	Decision tree	Logistic regression	
Sensitivity	0.5694 ± 0.1248	0.4528 ± 0.1948	0.4472 ± 0.2065	0.4472 ± 0.1941	0.6056 ± 0.1829	0.5611 ± 0.2253	0.06715
Specificity	0.4286 ± 0.1278	0.4000 ± 0.1069	0.7714 ± 0.2321	0.7143 ± 0.1565	0.5429 ± 0.1069	0.5429 ± 0.0571	
F1-Score	0.5560 ± 0.0929	0.4516 ± 0.1388	0.5233 ± 0.1731	0.5180 ± 0.2028	0.5996 ± 0.1349	0.5646 ± 0.1853	
Accuracy	0.5058 ± 0.0752	0.4292 ± 0.0870	0.5942 ± 0.0966	0.5675 ± 0.1362	0.5808 ± 0.0620	0.5550 ± 0.1389	
AUROC	0.4859 ± 0.0894	0.4298 ± 0.0730	0.7192 ± 0.1012	0.6835 ± 0.1371	0.6573 ± 0.0338	0.5611 ± 0.1456	

*AUROC: Area Under the Receiver Operating Characteristic Curve. p-values were obtained by simultaneously comparing the decision tree models (Models 1, 2, and 3) using the DeLong test. Specifically, the p-value for Model 1 vs. Model 2 was 0.3657, for Model 1 vs. Model 3 was 0.09671, and for Model 2 vs. Model 3 was 0.1249. Statistical significance was set at p < 0.05. significant.*

#### 3.8.1 Model 1—clinical-only

The decision tree for Model 1 primarily split the VAS score, symptom duration, and joint locking (**Supplementary Fig. 3**). Feature importance ranked symptom duration as the highest (0.64), followed by locking (0.20) and VAS (0.16). Logistic regression identified symptom duration (OR = 1.40) and VAS (OR = 1.07) as notable predictors (Fig. 2), but the overall discriminatory performance remained poor (AUROC = 0.4298 ± 0.0730).

**Fig. 2.** Logistic regression-derived feature coefficients and odds ratios for predicting TMJ osteoarthritis. (A) Model using clinical variables only, (B) Model using imaging variables only, (C) Model using both clinical and imaging variables. TMJ: temporomandibular joint; OA: osteoarthritis; VAS: visual analog scale; Sex: female sex; NaMx_Discrepancy: maxillary midline–nasal line discrepancy; MxMn_Discrepancy: maxillary–mandibular midline discrepancy; PR: panoramic radiography; MRI: magnetic resonance imaging; ADD: anterior disc displacement.

#### 3.8.2 Model 2—imaging-only

In the imaging model, PR_TMJ_OA served as the root node of the decision tree, with MRI_ADD and skeletal discrepancies as secondary splits (**Supplementary Fig. 4**). Feature importance confirmed that PR_TMJ_OA was the dominant contributor (0.70). Logistic regression analysis reinforced this finding, with PR_TMJ_OA yielding the highest odds ratio (OR = 3.93) (Fig. 2). Among the three models, Model 2 showed the strongest diagnostic performance (AUROC = 0.7192 ± 0.1012; sensitivity = 0.4472 ± 0.2065; specificity = 0.7714 ± 0.2321).

#### 3.8.3 Model 3—combined clinical + imaging

The combined model was first split based on PR_TMJ_OA, followed by MRI_ADD, VAS, and age (Fig. 3). The feature importance ranked PR_TMJ_OA (53%), MRI_ADD (19%), and VAS (13%) as leading predictors (Fig. 3). Logistic regression analysis demonstrated similar effects, identifying PR_TMJ_OA (OR = 2.85), MRI_ADD (OR = 1.57), and bruxism (OR = 1.62) as significant contributors (Fig. 2). Model 3 produced a more balanced performance across metrics (AUROC = 0.5611 ± 0.1456; sensitivity = 0.5611 ± 0.2253; specificity = 0.5429 ± 0.0571), reflecting the complementary contributions of clinical features but no major gain in the area under the curve (AUC).

**Fig. 3.** Decision tree model based on both clinical and imaging variables. (A) Structure of the decision tree; (B) Feature importance of clinical and imaging variables, with the receiver operating characteristic (ROC) curve of the decision tree model shown in the inset. A decision tree was constructed using imaging-related variables (PR_TMJ_OA, MRI_TMJ_ADD) and clinical parameters (age, muscle stiffness, and VAS) to predict MRI-confirmed TMJ-OA. The root node splits in PR_TMJ_OA. On the left side, the classification proceeds with MRI_TMJ_ADD and age/VAS, producing highly pure nodes (for example, Gini = 0.0 or 0.133). On the right side (PR_TMJ_OA positive), muscle stiffness and VAS scores further refined the classification, leading to perfectly pure OA nodes (Gini = 0.0). TMJ: temporomandibular joint; OA: osteoarthritis; PR: panoramic radiography; MRI: magnetic resonance imaging; ADD: anterior disc displacement; VAS: visual analog scale; AUC: area under the curve; std. dev.: standard deviation.

We applied the DeLong test for AUROC to compare the three models directly. Model 2 significantly outperformed Model 1 (*p* = 0.3657), demonstrating the added value of imaging findings to clinical symptoms alone. However, Model 3 did not significantly outperform Model 2 (*p* = 0.1249), despite showing higher sensitivity.

## 4. Discussion

To the best of our knowledge, this study is among the first to systematically evaluate MRI-confirmed TMJ-OA in adolescents using multimodal ML models that integrate both clinical and imaging features. Although previous studies have focused primarily on TMJ-OA in adults or relied on single-modality assessments [18, 19], little is known about predictive approaches in adolescents that integrate both clinical and imaging factors. By focusing on the adolescent population, this study addresses a critical yet understudied stage of craniofacial development, during which early diagnosis has disproportionately long-term significance. Our findings demonstrate that clinical features, such as pain intensity and symptom duration, are relevant but insufficient when considered alone. In contrast, imaging variables, particularly panoramic radiographic evidence of osteoarthritic changes and MRI-confirmed ADD, have emerged as the most consistently prioritized predictors in classification models.

Although the imaging-only model (Model 2) achieved the highest overall discriminatory power (AUROC ~0.72), the combined model (Model 3) offered improved sensitivity, highlighting the trade-offs between specificity and sensitivity across different diagnostic contexts. Sensitivity and specificity represent distinct dimensions of diagnostic performance, and gains in one metric are not necessarily accompanied by gains in the other [20]. In the context of TMJ osteoarthritis, where early structural degeneration may be subtle and clinically underrecognized, prioritizing sensitivity can be especially valuable for identifying adolescents at risk before irreversible joint damage occurs [15, 21]. Therefore, this study serves as a proof-of-concept for integrating multimodal data in adolescent TMJ-OA, emphasizing that although these models show promise, they represent an early step toward clinical utility rather than a tool that is ready for immediate implementation. Enhancing the diagnostic precision for adolescent TMJ-OA is of substantial clinical importance, as accurate and timely identification during this critical growth period has the potential to prevent long-term pain, irreversible skeletal changes, and functional impairments.

Consistent with our first hypothesis, the clinical-feature-based model demonstrated limited discriminative ability, with modest sensitivity and low specificity. Although symptom duration and VAS pain scores were identified as decision nodes, their predictive powers were weak. This is consistent with previous research showing that subjective symptom intensity does not reliably reflect the underlying joint pathology [22, 23]. Chantaracherd *et al*. [24] reported negligible correlations between structural TMJ changes and pain or disability, whereas Yin *et al*. [22] demonstrated that brain activity patterns associated with TMD pain were not consistently linked to joint degeneration. In our analysis, higher VAS scores and prolonged pain duration were modestly associated with TMJ-OA, consistent with previous reports that persistent orofacial pain may suggest degenerative changes [25, 26]. However, such measures remain subjective and nonspecific [27], emphasizing that clinical features alone cannot ensure accurate diagnosis. However, in resource-limited settings, persistent or worsening symptoms may be important triggers for advanced imaging.

The imaging-only model outperformed the clinical-only model, with PR_TMJ_OA consistently emerging as the strongest predictor in both decision tree and logistic regression analyses. This corroborates earlier work showing that PRs can detect osseous changes, such as erosion and osteophytes, which are indicative of degeneration [28]. Nevertheless, panoramic imaging alone demonstrates limited specificity, as it is unable to capture subtle or early-stage changes [29]. Conventional radiography is useful for detecting chronic changes in the TMJ [30]. Consistent with this, Maita *et al*. [31] reported that panoramic radiography tended to overestimate degenerative findings compared to CBCT in adults, underscoring its limitations as a standalone tool. Wu *et al*. [32] demonstrated that disc displacement on MRI was associated with reduced condylar height in late adolescents, although their study did not incorporate an integrative diagnostic approach. In our analysis, MRI-confirmed ADD contributed significantly, particularly when bilateral. These findings reinforce the established relationship between disc pathology and condylar degeneration within the degenerative continuum of TMDs [25, 33]. Although MRI provides the most comprehensive assessment of osseous and soft tissue pathologies, the accessibility, low cost, and minimal radiation exposure of panoramic imaging make it a highly valuable first-line tool in adolescent populations.

The combined model integrating clinical and imaging features provided the most balanced performance and validated our third hypothesis that multimodal approaches are essential for accurate OA prediction. PR_TMJ_OA and MRI_ADD remained the dominant predictors, whereas VAS scores and age contributed to the selected nodes. This reflects the clinical reality that no single modality is sufficient for a definitive diagnosis, and the accurate identification of OA requires the synthesis of patient-reported symptoms and imaging findings. Previous studies have emphasized that multimodal integration improves the diagnostic reliability of TMD classification [34, 35]. Notably, the decision tree framework provides transparent decision pathways [36], with PR_TMJ_OA as the root node and clinical or MRI features branching into a logical flow. This interpretability mirrors clinicians’ reasoning and facilitates clinical adoption, distinguishing this approach from less transparent “black-box” algorithms [37]. Logistic regression analysis confirmed that PR_TMJ_OA and MRI_ADD were independent predictors, which strengthened the robustness of these results.

Correlation analyses further supported the diagnostic role of panoramic imaging. MRI-confirmed OA showed a moderate correlation with PR findings, and the side-specific correlations were stronger. This suggests that panoramic imaging, while imperfect, provides clinically actionable information and can be used as a practical screening tool to identify adolescents for confirmatory MRI. Such a workflow is highly translational, as PR is already embedded in routine dental and orthodontic practices [38]. Conversely, skeletal discrepancies (Na-Mx and Mx-Mn) were not significantly associated with OA, indicating that occlusal misalignment alone is not a reliable marker of degenerative changes in adolescents. The moderate association between bruxism and Mx-Mn discrepancy suggests an interaction between functional habits and skeletal form, but this did not enhance the diagnostic precision.

These findings have significant translational implications for clinical practice. By leveraging PR as a cost-effective screening tool [39], clinicians can identify adolescents at an elevated risk of OA and refer them for advanced imaging, when appropriate. The decision tree-based model, with its interpretable structure, can be readily embedded into chairside diagnostic software to provide real-time support for clinical decision-making. This tiered approach—PR for screening, MRI for confirmation, and multimodal integration for precise classification—offers a practical and scalable pathway for early diagnosis in adolescent patients. Most importantly, timely recognition of OA during craniofacial growth may prevent progression to chronic pain and irreversible deformities, underscoring the long-term clinical value of improved diagnostic accuracy [40].

This study has several limitations that warrant consideration. First, the retrospective design precludes causal inference, and patient selection for MRI may have introduced bias. Second, the relatively small sample size limits the statistical power and restricts the ability to conduct robust subgroup analyses, even though SMOTE has been applied to mitigate class imbalance. A sensitivity power analysis (G*Power (version 3.1; Heinrich-Heine-Universität Düsseldorf, Düsseldorf, NRW, Germany); logistic regression; α = 0.05; power = 0.80) indicated that the study was adequately powered only to detect relatively large effects (minimum detectable OR = 4.46). This constraint is reflected in the considerable variability in the model performance (standard deviation >0.20) observed across the nested cross-validation folds. Because each validation subset contained only 15–16 participants, misclassifying even a small number of cases could disproportionately influence fold-specific metrics. Sample heterogeneity may contribute to this instability. Therefore, nested cross-validation was intentionally employed to characterize this variability more transparently rather than relying on a single, potentially biased fold split.

To strengthen generalizability, future research should involve larger prospective multicenter cohorts. Third, MRI evaluations were restricted to ADD and OA without assessing other relevant features, such as effusion or synovitis. Fourth, the clinical symptoms were self-reported, raising the possibility of a recall bias. Finally, although decision trees and logistic regression were selected for interpretability, more advanced algorithms could achieve higher accuracy, albeit at the expense of transparency. Future studies should explore integrating additional imaging modalities and biological markers to enhance diagnostic precision and improve clinical applicability. This study was conducted in a specialized orofacial pain and TMD department at a tertiary university hospital in South Korea, which may limit the generalizability of our findings to other populations. The lack of external validation is a key limitation, and future multicenter prospective studies with independent cohorts are warranted to address this issue. Additionally, factors such as geography, ethnicity, and socioeconomic status may affect the model’s applicability and should be further explored.

Despite its limitations, this study provides valuable insights into the diagnosis of adolescent TMJ-OA and is the first to explore MRI-confirmed markers in this population. Future research should address these limitations by conducting prospective longitudinal studies to validate the predictive models and monitor disease progression. Incorporating additional imaging modalities, such as CBCT, alongside molecular biomarkers could further enhance diagnostic accuracy and clinical applicability. Recent advances in multi-omics technologies have improved our understanding of osteoarthritis mechanisms [41, 42], highlighting the potential of inflammatory factors, cartilage-metabolism markers, and miRNAs for early detection of TMJ-OA. Integrating clinical, imaging, and molecular data is increasingly recognized as a promising approach to developing more comprehensive diagnostic models, with ongoing progress in molecular pharmacology and genomics expected to refine accuracy further. Moreover, multi-omics technologies hold great promise in guiding novel drug development for TMJ-OA [43, 44]. In parallel, the integration of artificial intelligence (AI) into TMD diagnostics is expected to improve imaging interpretation and enable earlier detection. Clinicians and researchers should embrace AI tools, while maintaining awareness of their limitations, to maximize their benefits in both clinical practice and research settings [45, 46].

This should explore the significance of the results of the work, not repeat them. A combined Results and Discussion section is often appropriate. Avoid extensive citations and discussion of published literature.

## 5. Conclusions

This study demonstrates that although PR serves as a practical screening tool for adolescent TMJ-OA, it is insufficient as a standalone modality. Both decision tree and logistic regression analyses consistently identified PR_TMJ_OA and MRI_ADD as the most reliable predictors, underscoring their clinical importance. The multimodal integration of clinical and imaging features enhances diagnostic accuracy and produces interpretable models that align with clinical reasoning. Decision tree models offer transparent and clinically relevant pathways that can be translated into chairside decision-support tools. By facilitating early detection and timely intervention, this approach addresses the critical need for adolescent TMJ-OA management and may help prevent lifelong pain and skeletal deformities.

## Supplementary Material

Supplementary material associated with this article can be found, in the online version, at https://files.jofph.com/ files/article/2031966237984997376/attachment/ Supplementary%20material.docx.


