# Supplementary material for: Decoding adolescent TMJ osteoarthritis with multimodal machine learning
Source: J Oral Facial Pain Headache. 2026 Mar 12;40(2):64–74. doi: 10.22514/jofph.2026.021 (PMC13036619; doi:10.22514/jofph.2026.021)
Supplement: Supplementary file 1 [file Supplementary-material.docx]

Supplementary material

Supplementary Table 1. Distribution of TMJ OA Laterality on MRI according to pain side, disc displacement, and panoramic radiographic OA.

| Parameter | Affected side | TMJ OA on MRI | | | | *p*-value |
| --- | --- | --- | --- | --- | --- | --- |
|  |  | Non-TMJ OA | TMJ OA (Right) | TMJ OA (Left) | TMJ OA (Bilateral) |  |
| Pain side^a^ | | | | | | |
|  | Right | 40.0% | 23.1% | 27.8% | 30.8% | 0.404 |
|  | Left | 22.9% | 38.5% | 27.8% | 23.1% |  |
|  | Bilateral | 37.1% | 38.5% | 44.4% | 46.2% |  |
| ADD on MRI^b^ | | | | | | |
|  | None | 28.6% | 23.1% | 16.7% | 0.0% | **0.003**** |
|  | Right | 20.0% | 7.7% | 0.0% | 15.4% |  |
|  | Left | 17.1% | 0.0% | 16.7% | 7.7% |  |
|  | Bilateral | 34.3% | 69.2% | 66.7% | 76.9% |  |
| TMJ OA on PR^b^ | | | | | | |
|  | None | 97.1% | 69.2% | 77.8% | 30.8% | **<0.001***** |
|  | Right | 2.9% | 23.1% | 0.0% | 0.0% |  |
|  | Left | 0.0% | 7.7% | 22.2% | 7.7% |  |
|  | Bilateral | 0.0% | 0.0% | 0.0% | 61.5% |  |

^a^Results were analyzed using the chi-square (χ²) test between two TMJ OA groups, also adjusted using the Bonferroni method. ^b^Results were obtained using Fisher’s exact test with Bonferroni correction. To obtain significant results, the two-tailed level of statistical significance was set at *p* < 0.05. ***p* < 0.01, ****p* < 0.001. Statistically significant values (*p* < 0.05) are shown in bold.

TMJ: temporomandibular joint; OA: osteoarthritis; ADD: anterior disc displacement; PR: panoramic radiography; MRI: magnetic resonance imaging.

Supplementary Table 2. Distinctive imaging features identified by MRI and panoramic radiography.

| Parameter | | Non-TMJ OA  (n = 35)  mean ± SD or n (%) | TMJ OA  (n = 44)  mean ± SD or n (%) | *p*-value |
| --- | --- | --- | --- | --- |
| MRI findings | | | | |
| MRI_TMJ OA^a^ | | | | |
|  | None | 35 (100.0%) | 0 (0.0%) | **<0.001***** |
|  | Right | 0 (0.0%) | 13 (29.5%) |  |
|  | Left | 0 (0.0%) | 18 (40.9%) |  |
|  | Bilateral | 0 (0.0%) | 13 (29.5%) |  |
| MRI_TMJ ADD^b^ | | | | |
|  | None | 10 (28.6%) | 6 (13.6%) | **0.015*** |
|  | Right | 7 (20.0%) | 3 (6.8%) |  |
|  | Left | 6 (17.1%) | 4 (9.1%) |  |
|  | Bilateral | 12 (34.3%) | 31 (70.5%) |  |
| Panoramic radiographic findings | | | | |
| PR_TMJ OA^a^ | | | | |
|  | None | 34 (97.1%) | 27 (61.4%) | **<0.001***** |
|  | Right | 1 (2.9%) | 3 (6.8%) |  |
|  | Left | 0 (0.0%) | 6 (13.6%) |  |
|  | Bilateral | 0 (0.0%) | 8 (18.2%) |  |
| NaMx discrepancy^b^ | | | | |
|  | (Absence) |  |  | 1.000 |
|  | (Presence) | 23 (65.7%) | 28 (63.6%) |  |
| MxMn discrepancy^b^ | | | | |
|  | (Absence) | 21 (60.0%) | 28 (63.6%) | 0.817 |
|  | (Presence) | 14 (40.0%) | 16 (36.4%) |  |

^a^Results were obtained using Fisher’s exact test with Bonferroni correction. ^b^Results were analyzed using the chi-square (χ²) test between two TMJ OA groups, also adjusted using the Bonferroni method. To obtain significant results, the two-tailed level of statistical significance was set at *p* < 0.05. **p* < 0.05, ****p* < 0.001. Statistically significant values (*p* < 0.05) are shown in bold. TMJ: temporomandibular joint; OA: osteoarthritis; ADD: anterior disc displacement; PR: panoramic radiography; MRI: Magnetic resonance imaging; SD: Standard Deviation.

Supplementary Table 3. Detailed results of a nested 5-fold cross validation for each logistic regression model.

| Parameter | Model 1 (only clinical data) | | Model 2 (only imaging data) | | Model 3 (clinical + imaging data) | |
| --- | --- | --- | --- | --- | --- | --- |
|  | AUROC | Accuracy | AUROC | Accuracy | AUROC | Accuracy |
| Test Fold 1 | 0.3651 | 0.3750 | 0.5476 | 0.4375 | 0.3810 | 0.3750 |
| Test Fold 2 | 0.4762 | 0.5000 | 0.6349 | 0.5625 | 0.6349 | 0.6875 |
| Test Fold 3 | 0.5397 | 0.5625 | 0.8730 | 0.7500 | 0.7302 | 0.6875 |
| Test Fold 4 | 0.4286 | 0.3750 | 0.8175 | 0.6875 | 0.6667 | 0.6250 |
| Test Fold 5 | 0.3393 | 0.3333 | 0.5446 | 0.4000 | 0.3929 | 0.4000 |

We report the AUROC and Accuracy of the Logistic Regression model for each 5 test folds. AUROC: area under the receiver operating characteristic curve.

Supplementary Table 4. Detailed results of a nested 5-fold cross validation for each tree model.

| Parameter | Model 1 (only clinical data) | | Model 2 (only imaging data) | | Model 3 (clinical + imaging data) | |
| --- | --- | --- | --- | --- | --- | --- |
|  | AUROC | Accuracy | AUROC | Accuracy | AUROC | Accuracy |
| Test Fold 1 | 0.6349 | 0.6250 | 0.5556 | 0.4375 | 0.6270 | 0.6250 |
| Test Fold 2 | 0.5159 | 0.4375 | 0.7619 | 0.6250 | 0.6508 | 0.5625 |
| Test Fold 3 | 0.4365 | 0.4375 | 0.8254 | 0.6875 | 0.7222 | 0.6250 |
| Test Fold 4 | 0.4762 | 0.5625 | 0.8016 | 0.6875 | 0.6349 | 0.6250 |
| Test Fold 5 | 0.3661 | 0.4667 | 0.6518 | 0.5333 | 0.6518 | 0.4667 |

We report the AUROC and Accuracy of the Tree model for each 5 test folds. AUROC: area under the receiver operating characteristic curve.

**
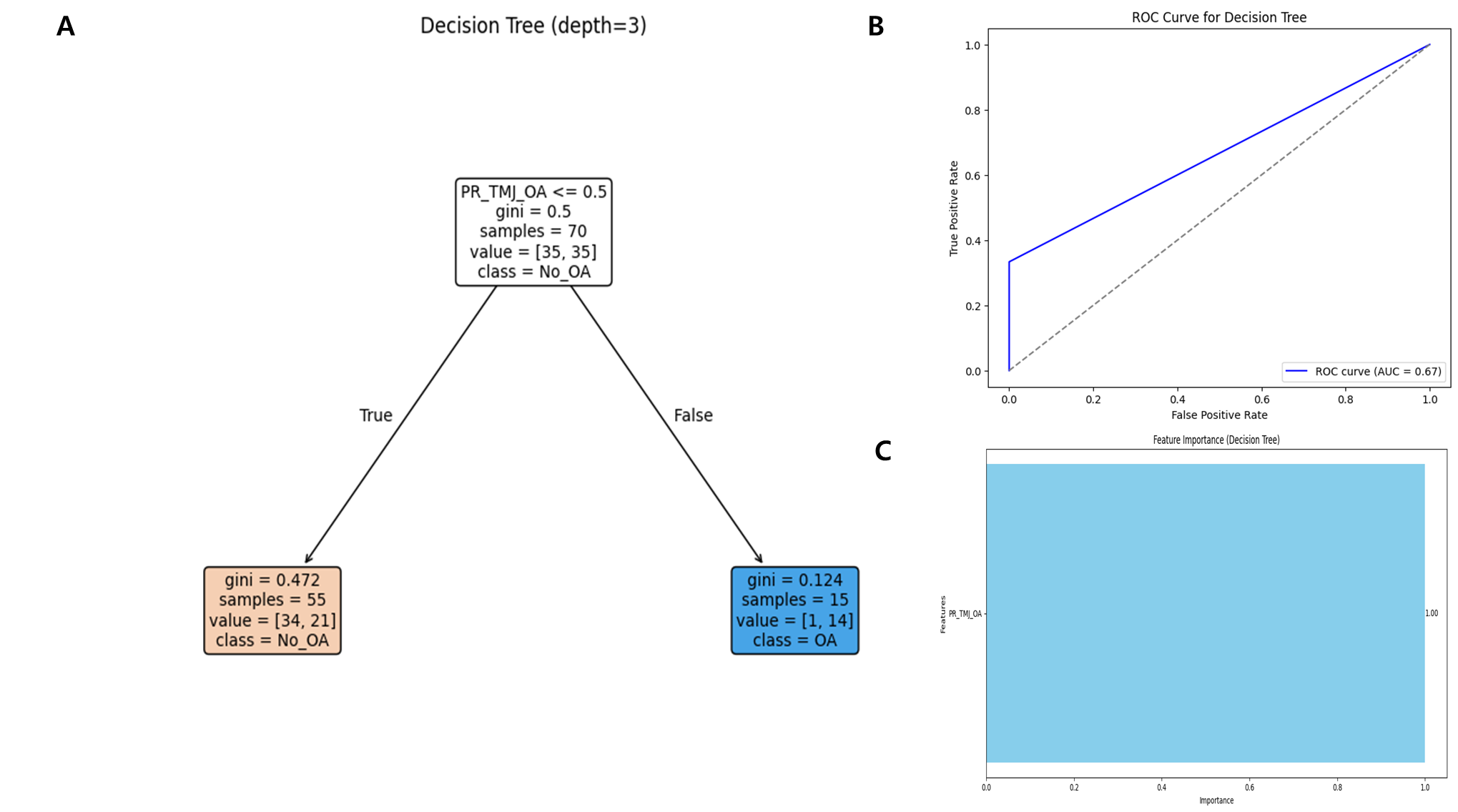
**

**Supplementary Fig. 1. Decision tree model for predicting MRI-confirmed TMJ osteoarthritis using panoramic radiographic OA alone.** (A) Structure of the decision tree; (B) Receiver operating characteristic (ROC) curve of the decision tree model; (C) Feature importance derived from imaging variables. This decision tree uses a single variable, PR_TMJ_OA (panoramic radiographic evidence of TMJ osteoarthritis), to classify MRI-confirmed TMJ OA. At the root node, if PR_TMJ_OA is ≤0.5, the majority of cases are classified as No_OA, with a Gini index of 0.472, indicating a moderate degree of class mixing. In contrast, when PR_TMJ_OA is >0.5, nearly all cases are classified as OA, and the Gini index drops to 0.124, reflecting high classification purity. TMJ: temporomandibular joint; OA: osteoarthritis; PR: panoramic radiography; ROC: receiver operating characteristic, AUC: area under the curve.


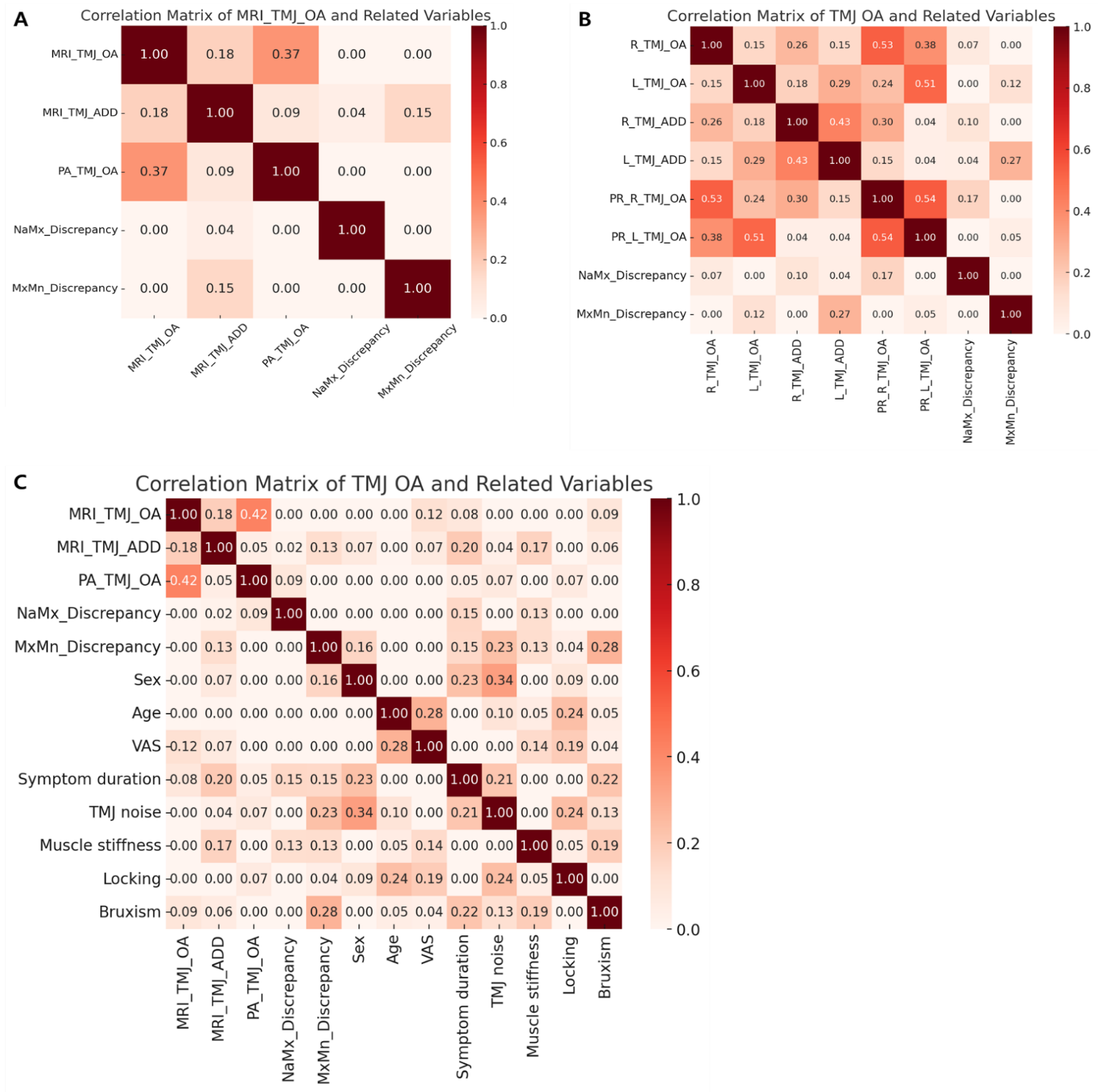


**Supplementary Fig. 2. Correlation matrices of TMJ OA with MRI findings, panoramic radiographic OA, and clinical variables.** (A) Correlation matrix between MRI-confirmed TMJ OA and other imaging-related features, (B) Correlation matrix stratified by laterality (right and left sides), (C) Correlation matrix including both clinical and imaging variables. TMJ: temporomandibular joint; OA: osteoarthritis; ADD: anterior disc displacement; MRI: magnetic resonance imaging, MRI-confirmed TMJ OA; MRI_TMJ_ADD: MRI-confirmed TMJ ADD; PA_TMJ_OA: panoramic radiographic TMJ OA; R_TMJ_OA: right-sided TMJ OA (on MRI); L_TMJ_OA: left-sided TMJ OA (on MRI); R_TMJ_ADD: right-sided TMJ ADD (on MRI); L_TMJ_ADD: left-sided TMJ ADD(on MRI); PR: panoramic radiography; PR_R_TMJ_OA: right-sided TMJ OA on PR; PR_L_TMJ_OA: left-sided TMJ OA on PR; NaMx_Discrepancy: maxillary midline–nasal line discrepancy; MxMn_Discrepancy: maxillary–mandibular midline discrepancy; VAS: visual analog scale for pain intensity; Symptom duration: duration of TMJ symptoms (in months).

**
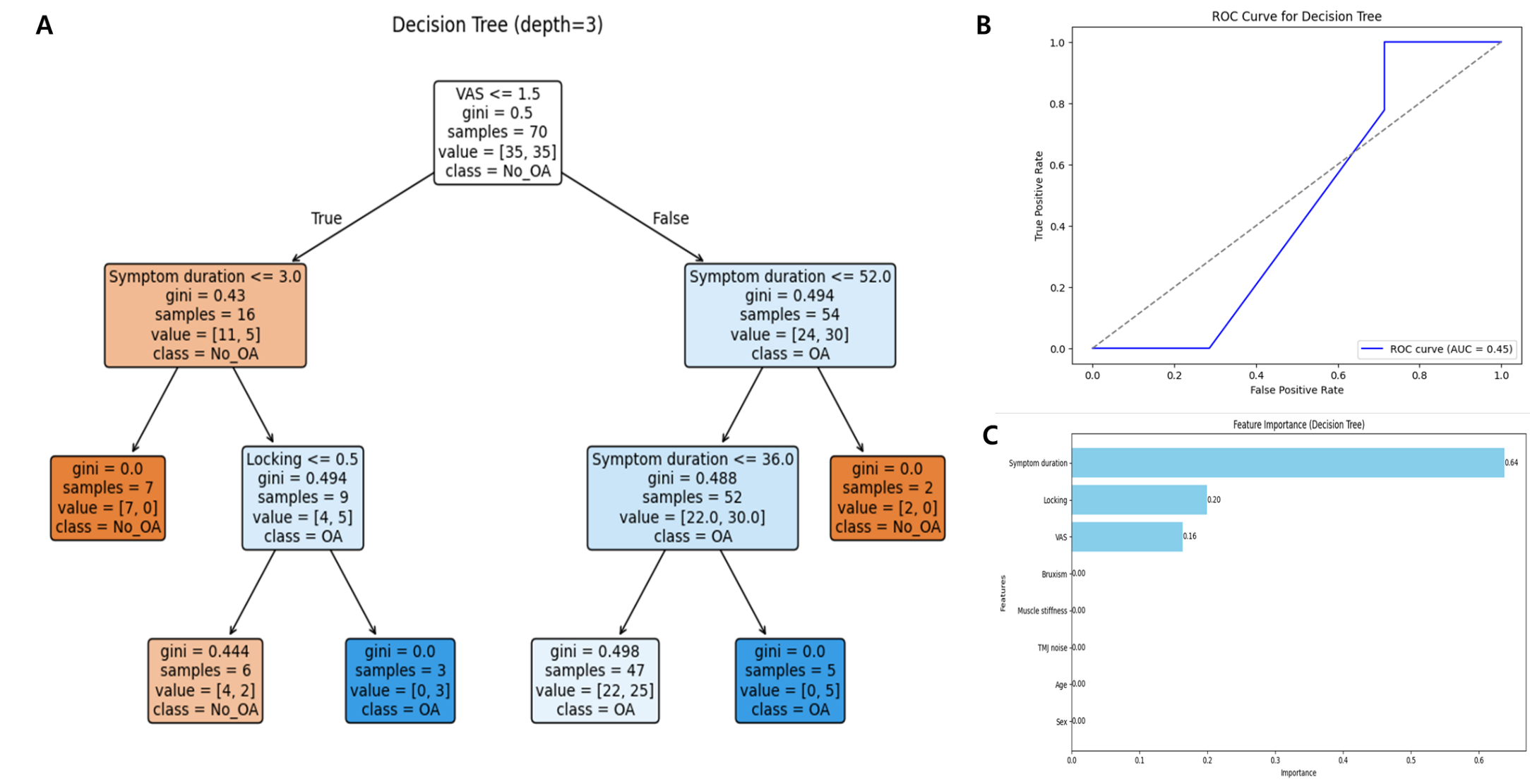
**

**Supplementary Fig. 3. Decision tree model based on clinical variables.** (A) Structure of the decision tree; (B) Receiver operating characteristic (ROC) curve of the decision tree model; (C) Feature importance derived from the model. Decision tree built using clinical variables such as VAS, symptom duration, and TMJ locking. The root node splits on VAS, and subsequent branches include symptom duration thresholds and locking presence. Nodes with perfect classification (Gini = 0.0) appear on both OA and No_OA sides, although several terminal nodes show class mixing (*e.g.*, Gini = 0.498). VAS: visual analog scale, OA: osteoarthritis, ROC: receiver operating characteristic; AUC: area under the curve.

**
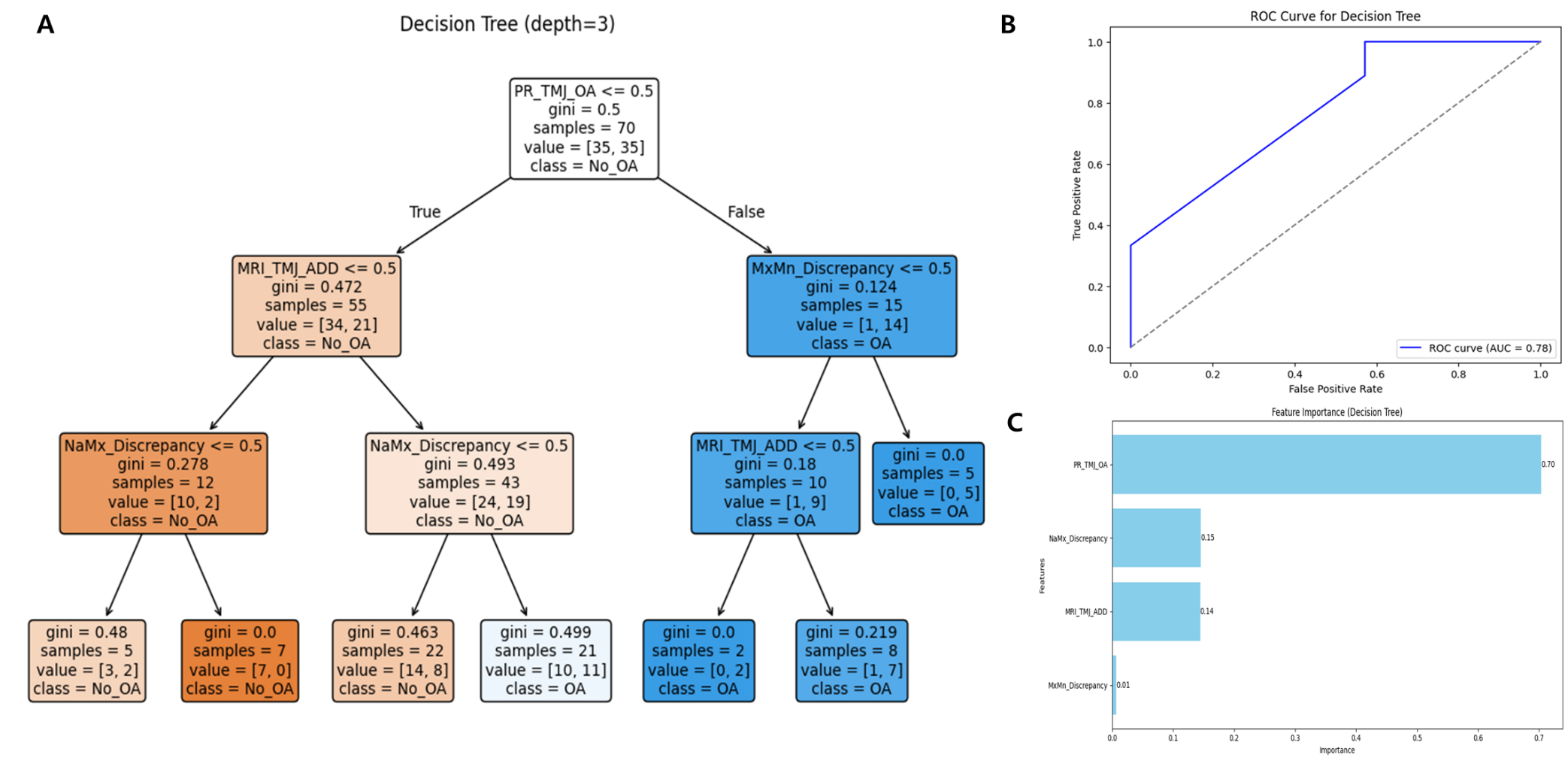
**

**Supplementary Fig. 4. Decision tree model based on MRI and panoramic radiographic imaging variables.** (A) Structure of the decision tree; (B) Receiver operating characteristic (ROC) curve of the decision tree model; (C) Feature importance derived from imaging variables. Decision tree constructed using imaging-related variables (PR_TMJ_OA, MRI_TMJ_ADD) and occlusal/clinical parameters (*e.g.*, age, muscle stiffness, VAS) to predict MRI-confirmed TMJ OA. The root node splits on PR_TMJ_OA. On the left side, classification proceeds with MRI_TMJ_ADD and age/VAS, producing some highly pure nodes (*e.g.*, Gini = 0.0 or 0.133). On the right side (PR_TMJ_OA positive), muscle stiffness and VAS further refine the classification, also leading to perfectly pure OA nodes (Gini = 0.0). TMJ: temporomandibular joint; OA: osteoarthritis; PR: panoramic radiography; MRI: magnetic resonance imaging; ADD: anterior disc displacement; ROC: receiver operating characteristic; AUC: area under the curve.
